# Digital Mental Health Interventions for Depression: Scoping Review of User Engagement

**DOI:** 10.2196/39204

**Published:** 2022-10-14

**Authors:** Jessica M Lipschitz, Rachel Van Boxtel, John Torous, Joseph Firth, Julia G Lebovitz, Katherine E Burdick, Timothy P Hogan

**Affiliations:** 1 Department of Psychiatry Brigham and Women's Hospital Boston, MA United States; 2 Department of Psychiatry Harvard Medical School Boston, MA United States; 3 Department of Psychiatry Beth Israel Deaconess Medical Center Boston, MA United States; 4 Division of Psychology and Mental Health University of Manchester Manchester Academic Health Science Centre Manchester United Kingdom; 5 Greater Manchester Mental Health NHS Foundation Trust Manchester Academic Health Science Centre Manchester United Kingdom; 6 Center for Healthcare Organization and Implementation Research Veterans Affairs Bedford Healthcare System Bedford, MA United States; 7 Department of Population and Data Sciences University of Texas Southwestern Medical Center Dallas, TX United States

**Keywords:** mHealth, mobile apps, engagement, adherence, randomized controlled trials, depression

## Abstract

**Background:**

While many digital mental health interventions (DMHIs) have been found to be efficacious, patient engagement with DMHIs has increasingly emerged as a concern for implementation in real-world clinical settings. To address engagement, we must first understand what standard engagement levels are in the context of randomized controlled trials (RCTs) and how these compare with other treatments.

**Objective:**

This scoping review aims to examine the state of reporting on intervention engagement in RCTs of mobile app–based interventions intended to treat symptoms of depression. We sought to identify what engagement metrics are and are not routinely reported as well as what the metrics that are reported reflect about standard engagement levels.

**Methods:**

We conducted a systematic search of 7 databases to identify studies meeting our eligibility criteria, namely, RCTs that evaluated use of a mobile app–based intervention in adults, for which depressive symptoms were a primary outcome of interest. We then extracted 2 kinds of information from each article: intervention details and indices of DMHI engagement. A 5-element framework of minimum necessary DMHI engagement reporting was derived by our team and guided our data extraction. This framework included (1) recommended app use as communicated to participants at enrollment and, when reported, app adherence criteria; (2) rate of intervention uptake among those assigned to the intervention; (3) level of app use metrics reported, specifically number of uses and time spent using the app; (4) duration of app use metrics (ie, weekly use patterns); and (5) number of intervention completers.

**Results:**

Database searching yielded 2083 unique records. Of these, 22 studies were eligible for inclusion. Only 64% (14/22) of studies included in this review specified rate of intervention uptake. Level of use metrics was only reported in 59% (13/22) of the studies reviewed. Approximately one-quarter of the studies (5/22, 23%) reported duration of use metrics. Only half (11/22, 50%) of the studies reported the number of participants who completed the app-based components of the intervention as intended or other metrics related to completion. Findings in those studies reporting metrics related to intervention completion indicated that between 14.4% and 93.0% of participants randomized to a DMHI condition completed the intervention as intended or according to a specified adherence criteria.

**Conclusions:**

Findings suggest that engagement was underreported and widely varied. It was not uncommon to see completion rates at or below 50% (11/22) of those participants randomized to a treatment condition or to simply see completion rates not reported at all. This variability in reporting suggests a failure to establish sufficient reporting standards and limits the conclusions that can be drawn about level of engagement with DMHIs. Based on these findings, the 5-element framework applied in this review may be useful as a minimum necessary standard for DMHI engagement reporting.

## Introduction

Digital mental health interventions (DMHIs) are a promising avenue for accessible treatment for people with widespread and debilitating mental health issues such as depression. The field of psychiatry continues to struggle with an insufficient supply of highly trained providers able to offer evidence-based services who are accessible in terms of location and cost. While face-to-face, evidence-based psychotherapy remains the first-line treatment option for mild to moderate depression [1], emerging literature on DMHIs suggests that these too could be an effective stand-alone or supplemental treatment option [2,3]. These interventions have, therefore, generated significant public interest as they are more accessible and lower cost than face-to-face psychotherapy.

As interest has mounted, however, so too have concerns about low patient engagement with these interventions. In the last 10 years, several large implementation studies of DMHIs have shown that the majority of patients offered these interventions do not engage at the recommended frequency or complete the full course of treatment [4-6]. In a large implementation study, Gilbody et al [7] concluded that “while [DMHIs] have been shown to be efficacious in developer led trials, [they were] not effective in usual NHS care settings. The main reason for this was low adherence and engagement with treatment rather than lack of efficacy.” Such low engagement rates threaten the clinical viability of these treatments.

DMHI engagement has been defined as a patient’s initial adoption and sustained interactions with an intervention [8-10]. Within the broader construct of engagement, intervention adherence refers to the extent to which participants engage in the content of the intervention as intended. In the context of randomized controlled trials (RCTs) intervention adherence can be reported as the number of intervention completers with the criteria for completion being clearly specified. However, within the broader construct of engagement, other metrics, such as the rate at which participants download and initiate intervention use (ie, uptake), degree or level of use of the intervention, and duration of use of the intervention are also relevant.

Engagement is particularly important to consider in RCTs because low intervention engagement poses a threat to the validity of conclusions drawn. It could lead to underestimating the intervention effect especially if a dose-response relationship exists [11]. Furthermore, as discussed by Eysenbach [12], if a participant did not significantly engage with an intervention, it is difficult to conclude that the intervention produced a positive outcome even if such outcomes were observed. In these cases, we are left with questions about the extent to which confounding variables, such as attention from study staff, could have produced any observed intervention effect. Finally, when degree of intervention engagement is not clearly described in manuscripts, we lose information on how an intervention must be used to achieve observed effects. For example, if an 8-week intervention period was studied and a positive intervention effect was observed, but 70% of participants only used the intervention for the first 2 weeks of the intervention period, we may conclude that just 2 weeks of use may be producing positive results. Alternatively, we may conclude that a certain level of effect could be expected after 2 weeks of use, whereas a different, perhaps more pronounced effect, could be expected after 8 weeks of use.

The concept of what constitutes sufficient engagement with DMHIs is inherently messier than for some other types of mental health interventions. For example, sufficient engagement with antidepressant medications typically means taking a daily pill. In psychotherapy, sufficient engagement is typically defined as attending all planned psychotherapy sessions. Use of medication and appointment attendance are clear quantitative adherence metrics. In the case of DMHIs, however, heterogeneity in intervention design leaves us with considerably less clarity on appropriate intervention adherence metrics. Some DMHIs, such as the Get Happy Program [13], consist of a series of lessons or modules that are designed to be completed in a sequential fashion over a specified number of weeks. These programs mirror face-to-face therapy programs where there is an assumption of some established weekly content review or dedicated time commitment. Other DMHIs are designed to be used more frequently for briefer periods. For example, IntelliCare [14] is designed to be used on a daily basis, but length of time in the app is not prescribed. Still, other interventions (eg, the MONARCA System [15]) consist primarily of symptom monitoring and are designed to be used frequently to inform and support clinician-based care.

This inherent heterogeneity of DMHIs makes engagement difficult to compare across studies. It also calls for consideration of what constitutes appropriate reporting related to both the larger construct of engagement and the narrower construct of adherence. To date, reviews and meta-analyses related to engagement with DMHIs have tended to focus on related, but distinct concepts. For example, study dropout or study attrition has been evaluated as a proxy for treatment dropout, with findings suggesting significant dropout [16-18]. Similarly, user-rated acceptability and feasibility have been evaluated as proxies for engagement [19]. Finally, several recent reviews have explored variables related to user engagement with DMHIs [8,9,18]. However, to date, no review to our knowledge has explored the actual level of user engagement in RCTs of DMHIs. Therefore, the objective of this scoping review was to examine reporting on user engagement in RCTs of mobile app–based interventions for symptoms of depression. Specifically, we sought to identify (1) the extent to which key engagement metrics are routinely reported and (2) what the metrics that are reported reflect about standard levels of engagement.

## Methods

The creation of this report was guided by the PRISMA (Preferred Reporting Items for Systematic Reviews and Meta-Analyses) Extension for Scoping Reviews (Multimedia Appendix 1) [20].

### Information Sources and Search Strategy

A systematic search was conducted using OvidSP to search 7 electronic databases, MEDLINE, Embase, Cochrane Central Register of Controlled Trials, Allied and Complementary Medicine, Health Management Information Consortium, Health Technology Assessment, and PsycINFO, for articles published through May 1, 2020 (Table 1). The search was conducted on May 7, 2020. In brief, the search strategy combined synonyms for the population of interest (patients with mental illness), the intervention modality (mobile phone apps), and the type of study (RCT). Search results were limited to the English language and studies of humans.

**Table 1:** Search strategy as used in OvidSP on May 7, 2020.

Search category	Search terms
Population	“depression” OR “depressive” OR “mental illness” OR “mental health” OR “mood disorder” OR “affective disorder” OR “anxiety” OR “panic disorder” OR “phobia” OR “bipolar” OR “psychosis” OR “schizophr*” AND
Intervention	“smartphone*” OR “mobile phone*” OR “cell phone*” OR “iphone” OR “android” OR “mhealth” OR “mobile application” OR “phone application” AND
Type of study	“randomised” OR “randomized” OR “randomly” OR “random assignment” OR “controlled trial” OR “clinical trial” OR “control group” OR “intervention”
Platform used	OvidSP
Databases selected for search	Ovid MEDLINE, Embase, Cochrane Central Register of Controlled Trials, Allied and Complementary Medicine, Health Management Information Consortium, Health Technology Assessment, and PsycINFO

### Inclusion and Exclusion Criteria

Only articles published in peer-reviewed journals were included. Articles were deemed eligible if they were RCTs of mobile app–based interventions targeting adults (aged >18) with clinical depression, in which depressive symptoms were a primary outcome of interest, and retention in posttreatment assessments was reported. We defined a mobile app–based intervention as one that required use of a mobile device app as part of the treatment.

We defined studying a “clinically depressed” sample as meeting at least one of the following criteria: (1) eligibility criteria requiring participants to have scores on a depression self-report measure over an established clinical cutoff; (2) eligibility criteria requiring participants to have a psychiatric diagnosis per their medical record or per a structured clinical interview; or (3) reported average baseline scores on a depression self-report measure above an established clinical cutoff in all groups. When there was ambiguity on the established clinical cutoff for a self-report measure, we used the lowest published cutoff score.

At least two independent reviewers judged article eligibility (JML, JGL, or RVB), with any disagreements resolved through mediation with a third reviewer (TPH). The screening process began with title and abstract review followed by a full-text review of any articles that appeared potentially relevant based on the abstract/title review or where there was insufficient information in the abstract to determine eligibility.

### Data Extraction and Synthesis

Data extraction occurred in 3 parts. First, data were extracted by one author (JGL or RVB). Next, the rationale for each datapoint and where it came from in the original articles were reviewed with JML. Finally, all datapoints considered ambiguous or disagreements between the authors who completed the initial data extraction and JML were reviewed with one additional author (TPH).

Two kinds of information were extracted from each article. First, intervention details were extracted, including the (1) clinical population, (2) length of the treatment period, (3) a description of the study conditions, (4) total sample size in each condition, and (5) whether human support by a coach or licensed clinician was offered as part of the intervention.

Second, a 5-element framework of minimum necessary DMHI engagement reporting, developed by our study team, was used to extract key descriptive and numeric indices of participant engagement with the intervention. Elements in this framework were as follows: (1) recommended intervention app use as communicated to participants at enrollment and, when reported, intervention app adherence criteria; (2) rate of uptake, defined as the number and percentage of participants randomized to the intervention who engaged with their assigned app at all; (3) level of intervention app use metrics, specifically number of times participants used the app and amount of time participants spent in the app; (4) duration of intervention app use metrics (ie, whether weekly use patterns were reported and the number and percentage of participants who used the app in the final week of the intervention period); and (5) number and percentage of participants randomized who could be considered intervention completers. Furthermore, for context, we identified whether studies used backend data or other methods (such as self-report) to quantify app usage and extracted any additional data presented on intervention engagement.

## Results

### Selection and Inclusion of Studies

**Figure 1:**
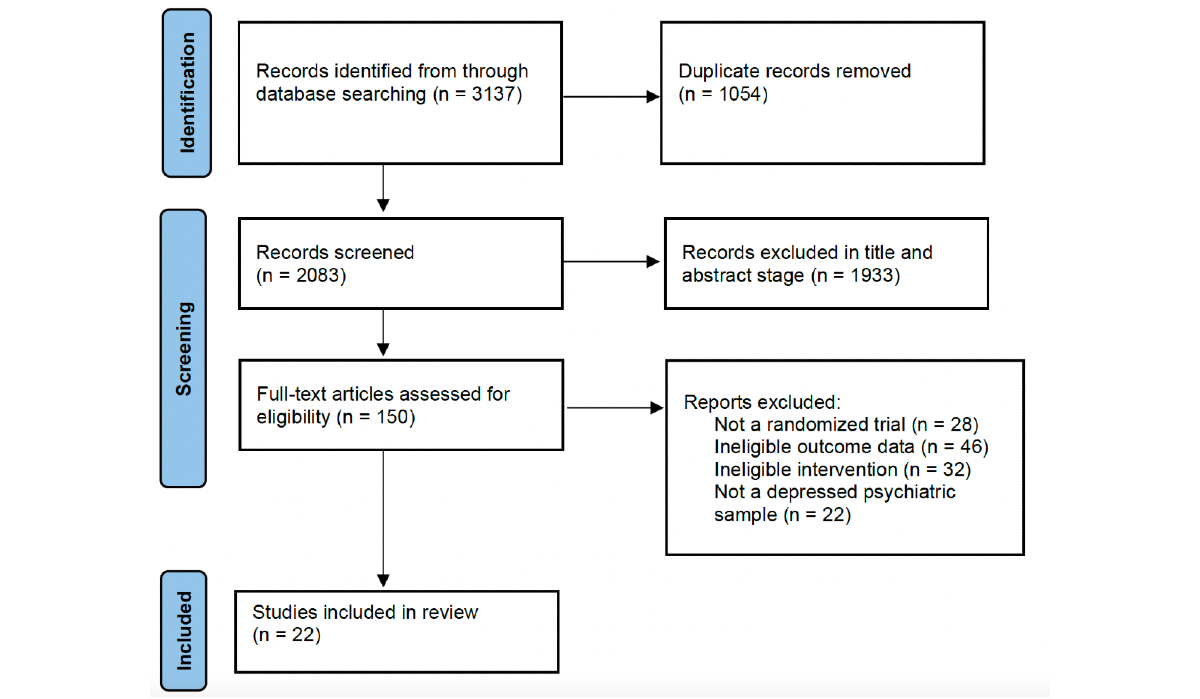
PRISMA Search Diagram.

The full systematic search retrieved a total of 3137 records (Figure 1). Following the removal of duplicate articles across electronic databases, 2083 articles were screened at the title-and-abstract phase. This identified 150 articles as potentially eligible, which were subsequently screened in full. Full-text screening resulted in the exclusion of 128 articles for reasons specified in Figure 1. A total of 22 independent studies [13,15,21-40] were ultimately eligible for inclusion.

### Characteristics of Included Studies

Detailed study characteristics are presented in Table 2. While all 22 studies included a clinically depressed sample and symptoms of depression as a primary outcome, the target populations differed. Of the 22 eligible studies, the following target populations were recruited: depression (n=13), suicidal ideation (n=1); depression or anxiety (n=3); bipolar disorder (n=1); medical population with clinically significant symptoms of depression (n=2); community sample (n=1); and college students (n=1). Intervention periods ranged from 2 weeks to 6 months and sample sizes ranged from 30 to 720. Interventions evaluated included a range of human support: 11 were entirely self-help interventions involving no human support, 9 involved a licensed clinician, 1 involved a clinical coach, and 1 included clinical support from research staff for whom licensure status was not specified. For descriptive purposes, apps studied were assigned to 1 of 3 categories: those intended to be used as daily self-management/skill-building tools (n=13); those intended to provide support in the context of clinician-administered care or to facilitate communication with clinicians (n=5); and treatments involving a discrete number of lessons/modules typically to be completed on a weekly basis (n=4).

**Table 2:** Study characteristics.

Study	Clinical population	Treatment period	Conditions	Sample size	Human contact	App category
Arean et al [21]	Depression	4 weeks	Project: EVO (gamified cognitive training app) iPST (problem-solving therapy app) Health Tips (app providing daily tips for improved health; control)	209 211 206	None None None	Daily self-management/skill building
Bakker et al [22]	Community sample	30 days	MoodKit (CBT^a^-based app with a variety of tools) MoodPrism (self-monitoring mood-tracking app) MoodMission (CBT-based app that recommends CBT strategies in response to user-reported low moods and anxious feelings) Waitlist (control)	78 78 78 78	None None None None	Daily self-management/skill building
Birney et al [23]	Depression	6 weeks	MoodHacker (CBT-based depression management app based on the “Coping with Depression” program) Alternate care group (emailed links to 6 websites with depression information; control)	150 150	None None	Daily self-management/skill building
Borjalilu et al [24]	College students	20 days 6 weeks 6 weeks	Aramgar stress management app (mindfulness-based stress reduction) Blended (Aramgar app for 20 days + 6 weeks of face-to-face therapy) Face-to-face therapy only	20 28 20	None Clinician Clinician	Daily self-management/skill building
Dahne et al [25]	Depression	8 weeks	¡Apívate! (Spanish language brief behavioral activation mobile app) iCouch CBT (Spanish language CBT mobile app; active control) TAU^b^ (control)	22 9 11	None None N/A^c^	Daily self-management/skill building
Dahne et al [26]	Depression	8 weeks	Moodivate (brief behavioral activation mobile app) MoodKit (CBT mobile app; active control) TAU (control)	24 19 9	None None N/A	Daily self-management/skill building
Faurholt-Jepsen et al [15]	Bipolar disorder	6 months	MONARCA system (daily self-monitoring app with feedback from clinician) Placebo MONARCA (Android cellphone and TAU; control)	39 39	Clinician Clinician	Support for appointments/interaction with clinician
Fitzpatrick et al [27]	Depression or anxiety	2 weeks	Woebot (CBT-oriented conversational agent app) “Depression in College Students” eBook created by the National Institute of Mental Health (informational booklet; control)	34 36	None None	Daily self-management/skill building
Guo et al [28]	Patients with HIV with depression symptoms	3 months	Run4Love (WeChat-based cognitive behavioral stress management course plus physical activity promotion) Usual care (brochure on nutrition and usual care for HIV; control)	150 150	Study staff (unclear if coach/clinician) None	Discrete number of lessons/modules
Lüdtke et al [29]	Depression	4 weeks	Be Good to Yourself app (40 self-help strategies and exercises, based on CBT) Waitlist (control)	44 44	None None	Daily self-management/skill building
Ly et al [30]	Depression	8 weeks	Behavioral activation smartphone app Mindfulness smartphone app	40 41	Clinician Clinician	Support for appointments/interaction with clinician^d^
Ly et al [31]	Depression	9 weeks 10 weeks	Blended treatment (4 face-to-face behavioral activation sessions plus a smartphone app for support and suggestions between sessions) Full behavioral activation (10 face-to-face behavioral activation sessions; control)	46 47	Clinician Clinician	Support for appointments/interaction with clinician
Mantani et al [32]	Depression	9 weeks	Kokoro app (8 sessions, CBT-based self-help app + antidepressant switch) Antidepressant switch (control)	81 83	Clinician Clinician	Discrete number of lessons/modules
Moberg et al [33]	Depression or anxiety	1 month	Pacifica (guided CBT-based self-help app) Waitlist (control)	253 247	None None	Daily self-management/skill building
Mohr et al [34]	Depression or anxiety	8 weeks	IntelliCare platform with coach (12 apps, each focusing on a single psychological or behavioral strategy) IntelliCare platform self-guided IntelliCare platform with recommendations IntelliCare platform no recommendations	150^e^ 151 149 152	Coached None Half coached Half coached	Daily self-management/skill building
Motter et al [35]	Depression	8 weeks	Executive function/processing speed focused–computerized cognitive training Verbal ability–focused computerized cognitive training	25 21	None None	Daily self-management/skill building
O’Toole et al [36]	Patients referred for suicidal thoughts	About 8 weeks (range 8-16 weeks, at clinician discretion)	LifeApp’tite mobile app (suicide prevention app provided alongside suicide prevention psychotherapy protocol) TAU (suicide prevention psychotherapy protocol; control)	60 69	Clinician Clinician	Support for appointments/interaction with clinician
Place et al [37]	Primary care behavioral health patients	6 months	Usual care (in behavioral health clinic) + Cogito’s mobile sensing platform Usual Care (control)	35 33	Clinician Clinician	Support for appointments/interaction with clinician
Proudfoot et al [40]	Depression	7 weeks	MyCompass Intervention (app with 12 skill-building modules derived from CBT, interpersonal psychotherapy, problem-solving therapy, and positive psychology) Attention control (weekly mental health fact sheet delivered to email inbox; active control) Waitlist (control)	242 248 230	None None None	Discrete number of lessons/modules
Roepke et al [38]^f^	Depression	4 weeks	CBT-PPT SB (SuperBetter game–like app with additional content from cognitive behavioral therapy and positive psychotherapy) General SB (SuperBetter game–like app with additional content focused on self-esteem and acceptance) Waitlist (control)	93 97 93	None None N/A	Daily self-management/skill building
Stiles-Shields et al [39]	Depression	6 weeks	Boost Me (an app intervention based on activity scheduling) Thought Challenger (an app intervention based on thought restructuring) Waitlist control (control)	10 10 10	Cliniciang Clinician N/A	Daily self-management/skill building
Watts et al [13]	Depression	8 weeks	Get Happy Program mobile app (6 lessons on how to manage depression symptoms) Get Happy Program computer delivered	22 30	Clinician Clinician	Discrete number of lessons/modules

^a^CBT: cognitive behavioral therapy.

^b^TAU: treatment as usual.

^c^N/A: no treatment administered.

^d^The intervention in Ly et al [30] contained elements of daily self-management/skill building, but completion was defined by interactions with a clinician so this was deemed primarily an intervention to support appointments/interaction with a clinician.

^e^Mohr et al [34] was a 2 × 2 factorial trial design. Group sample sizes specified here are not mutually exclusive.

^f^Roepke et al [38] reported that the SuperBetter intervention was targeted to occur on the iPhone, but could be used via a website on computers. This study was deemed eligible because the intention was for it to be smartphone based.

^g^Stiles-Shields et al [39] involved coaching, but is categorized as involving a clinician (not a coach) because the coach was a licensed clinician.

### Reporting on Participant Engagement

Data extracted based on our 5-element framework are presented in Table 3 (with additional details presented in Multimedia Appendix 2). With the exception of Ludtke et al [29], all studies that reported on app usage indicated using backend data from the app to monitor app usage in the test condition(s). Ludtke et al [29] only offered self-reported app usage data; 14/22 papers (64%) reported the rate of app uptake defined as the number of participants randomized to the intervention condition(s) who engaged with the app at least once. Findings in those studies reporting the rate of app uptake indicated that between 42% and 100% of those participants randomized to an app-based DMHI condition engaged with the app at least once.

With regard to ongoing use, reports were varied. A total of 13 papers (59%) reported a level-of-use metric. The most common level-of-used metric was number of sessions/launches (n=12). Time spent in the app was a less popular level-of-use metric (n=4). Fewer papers reported metrics on duration of use. Only 5 studies (23%) reported weekly use patterns over the course of the intervention and the number of participants who were still using the intervention during the last week of the treatment period.

With regard to questions of whether participants completed the intervention as intended, reporting was also varied. Table 3 describes the app intervention instructions given to participants and app adherence criteria to the extent that these were specified in each article. Only 3 studies clearly reported the number of participants randomized to the DMHI who were considered to have completed the app-based components of the intervention as intended per specified intervention instructions. An additional 4 studies (footnote i in Table 3) reported the number of participants who met a specified adherence threshold such as using the intervention app once per week; 4 more studies reported metrics related to intervention completion, including percentage of patients who used the app on a daily basis (n=1; footnote m); percentage of patients completing the intervention based on a criterion defined by clinician contact rather than app use (n=2; marked by footnote o); and percentage of participants who downloaded all the intervention content (n=1; marked by footnote t). Findings in those studies reporting metrics related to intervention completion indicated that between 14.4% and 93.0% of participants randomized to a DMHI condition completed the intervention as intended or according to a specified app adherence criteria. Among the 11 studies reporting this metric, 6 reported that less than or equal to 50% of participants completed the intervention.

**Table 3:** Treatment engagement metrics for digital mental health interventions.^a^

Study and intervention name			App use instructions or adherence criteria		Rate of uptake^b^, n (%)		Level of use				Duration of use					Completers^c^, n (%) or %		
							App uses		Minutes spent using the app, mean (SD)		Reported weekly use pattern		Used in the final week, n (%) or %					
**Arean et al [21]**																		
	Project: EVO	Use app 6 times/week for 30 minutes/day (3 or more times/week considered adherent)		177 (42.1)^d^		Mean 10.78 (SD 11.4)^e^		NR^f^		Yes		42 (20.1)^g^		30 (14.4)^g,h,i^				
	iPST	Use app as often as possible (1 or more times/week considered adherent)		—^j^		—^j^		NR		Yes		40 (19.0)^g^		40 (19.0)^g,h,i^				
	Health Tips App	No specific instructions, but daily advice was provided		NR		NR		NR		No		NR		NR				
**Bakker et al [22]**																		
	MoodKit	No specific instructions reported		NR		NR		NR		No		NR		NR				
	MoodPrism	No specific instructions reported		NR		NR		NR		No		NR		NR				
	MoodMission	No specific instructions reported		NR		NR		NR		No		NR		NR				
**Birney et al [23]**																		
	MoodHacker	Daily app use		NR		Mean 16.0 (SD 13.3)		78 (78)		No		NR		NR				
**Borjalilu et al [24]**																		
	Aramgar app	Complete recommended app exercises daily		NR		NR		NR		No		NR		NR				
	Aramgar app with face-to-face therapy	Twice/week face-to-face workshops plus daily app exercises		NR		NR		NR		No		NR		NR				
**Dahne et al [25]**																		
	¡Apívate!	Use app once/day (1 or more times/week considered adherent)		22 (100)		Mean 61.4 (SD 91.7)		65.8 (82.8)		Yes		11 (50)		11 (50)^h,i^				
	iCouch CBT	Use app once/day (1 or more times/week considered adherent)		NR		NR		NR		Yes		33^g^		33^g,h,i^				
**Dahne et al [26]**																		
	Moodivate	Use the app once/day (1 or more times/week considered adherent)		21 (100)^k^		Mean 46.8 (SD 30.1)		120.8 (101.0)		Yes		9 (50)^k^		9 (50)^h,i,k^				
	MoodKit	Use app once/day		NR		NR		NR		No		NR		NR				
**Faurholt-Jepsen et al [15]**																		
	MONARCA	Use app for self-monitoring daily		34 (87.2)^l^		NR		NR		No		NR		93.0^m^				
**Fitzpatrick et al [27]**																		
	Woebot	Daily monitoring and “regular check-ins”		34 (100)		Mean 12.14 (SD 2.23)		NR		No		NR		NR				
**Guo et al [28]**																		
	Run4Love	Complete 9 cognitive behavioral stress management sessions, 3 review sessions, and set weekly physical activity goal		NR		NR		NR		No		NR		NR				
**Lüdtke et al [29]**																		
	Be Good to Yourself app	Use app “several times a week”		26 (59.1)^n^		NR		NR		No		NR		19 (43.2)^n^				
**Ly et al [30]**																		
	Behavioral activation smartphone app	Add at least two behavioral goals to the app and register/write a reflection in the app when these goals were completed		81 (96.4)^d^		NR		NR		No		NR		25 (63.0)^e,o^				
	Mindfulness smartphone app	Use audio tracks with exercises to facilitate the practice of mindfulness		—^j^		—^j^		NR		No		NR		32 (78.0)^e,o^				
**Ly et al [31]**																		
	Blended treatment	No specific instructions reported		NR		NR		NR		No		NR		42 (91.3)^o^				
**Mantani et al [32]**																		
	CPT-Kokoro app	Complete 8 mobile app sessions, 1 per week		80 (98.76)		Mean 7.01 (SD 1.5)^p^		NR		Yes		43 (53.1)		43 (53.1)^p^				
**Moberg et al [33]**																		
	Pacifica	No specific instructions reported		246 (97.2)		Median 19 (range 1-286)^e^		NR		No		NR		NR				
**Mohr et al [34]**																		
	IntelliCare: coached	No specific instructions reported (last app use at or after 7 weeks considered adherent)		143 (95.3)^l^		Median 215 (IQR 141-330.8)		NR		Yes		136 (90.7)^q^		136 (90.7)^m,p^				
	IntelliCare: self-guided	No specific instructions reported (last app use at or after 7 weeks considered adherent)		151 (100)^l^		Median 218 (IQR 113-310)		NR		Yes		126 (83.4)^q^		126 (83.4)^m,p^				
	IntelliCare: recommendations	No specific instructions reported (last app use at or after 7 weeks considered adherent)		146 (98.0)^l^		Median 232 (IQR 126-356)		NR		Yes		132 (88.6)^q^		132 (88.6)^m,p^				
	IntelliCare: no recommendations	No specific instructions reported (last app use at or after 7 weeks considered adherent)		148 (97.4)^l^		Median 201.5 (IQR 125.8-285.5)		NR		Yes		130 (85.5)^q^		130 (85.5)^m,p^				
**Motter et al [35]**																		
	Executive function/processing speed–focused CCT^r^	Use app 15 minutes/day 5 days/week		NR		NR		168.3 (69.0)		No		NR		NR				
	Verbal ability–focused CCT	Use app 15 minutes/day 5 days/week		NR		NR		363.8 (253.4)		No		NR		NR				
**O’Toole et al [36]**																		
	LifeApp’tite	At discretion of therapists to decide frequency of app use		50 (83.3)		NR		NR		No		NR		NR				
**Place et al [37]**																		
	Cogito	Record weekly audio notes on mood and complete weekly self-reports		NR		NR		NR		No		NR		NR				
**Proudfoot et al [40]**																		
	MyCompass	Complete a minimum of 2 modules and monitor at least three moods or behaviors		NR		Mean 14.7 (SD 16.7)^p^		NR		No		NR		NR				
**Roepke et al [38]**																		
	CBT-PPT SuperBetter	Use app 10 minutes/day		72 (77.4)		Mean 21.5 (SD 34.3), median 9.5^d,e^		NR		No		NR		31 (33.3)^s^				
	General SuperBetter	Use app 10 minutes/day		72 (74.23)		—^j^		NR		No		NR		64 (66.0)^s^				
**Stiles-Shields et al [39]**																		
	Boost Me	No specific instructions reported		10 (100)^l^		Mean 97.7		NR		No		NR		NR				
	Thought Challenger	No specific instructions reported		7 (70)^l^		Mean 33.5		NR		No		NR		NR				
**Watts et al [13]**																		
	Get Happy Program Mobile App	Complete 6 lessons and associated homework		15 (68.2)^l^		Mean 5.1 (SD 1.6)^e,t^		NR		No^u^		NR^u^		10 (45.5)^p^				

^a^Table includes all treatment conditions that involved a mobile app component.

^b^Rate of uptake: number of participants randomized to the intervention who used it at least once.

^c^Completer: participants who completed the intervention as intended per intervention instructions or per specified adherence criteria.

^d^Reported metric cut across treatment groups.

^e^Only included participants who logged onto the app at least once.

^f^NR: not reported.

^g^Estimate based on figure, exact number not reported.

^h^Assumes participants who met adherence criteria during the last week also met adherence criteria in previous weeks. For example, in a 4-week intervention, those reported to have used the app in week 4 also used in weeks 1-3.

^i^Completion refers to meeting a specified adherence criteria involving app use not to complying with intervention use instructions.

^j^Metrics were only reported across conditions rather than for each group independently; all numbers are rounded to 1 decimal place.

^k^Owing to technical issues, data on rate of uptake were only available in 21 participants and data on ongoing use were only available in 18 participants. To calculate percentages presented, the number of people for whom data were available was used as the denominator.

^l^Based on reported numbers of participants who were randomized to the condition, but never started treatment. Reasons were not always related to willingness/interest in trying the relevant app. For example, reason may have been that the participant was unresponsive to outreach to inform them of their assigned treatment.

^m^Article reports “93.03% (SD 15.6) of patients randomized to the intervention group evaluated the subjective items in the MONARCA system on a daily basis.” Unclear if this refers to participants using the system an average of 93.03% of days or if it refers to 93.03% of the participants in the intervention using it every day of the 6-month intervention period.

^n^As use data were self-reported, these metrics only include those participants who completed the posttreatment assessment. To calculate percentages presented, the total size of the treatment group was used as the denominator.

^o^Completion was defined by clinician contact not app use.

^p^Metric takes into account all participants randomized to the condition even if they did not log onto the app.

^q^Number represents the number of participants whose last use was week 7 or after.

^r^CCT: computerized cognitive training.

^s^Refers to the number of participants who downloaded all content.

^t^Uses refers to lessons completed.

^u^Number of lessons completed was reported, but lessons were not precisely 1 per week.

## Discussion

### Principal Findings

This scoping review has revealed that reporting on engagement with DMHIs in RCTs is highly variable. A number of basic metrics of intervention engagement, such as rate of intervention uptake, weekly use patterns, and number of intervention completers, were routinely not reported. When intervention engagement metrics were reported, it was common to see low levels of engagement. The variability in reporting and frequency of low engagement when reported highlight the importance of establishing minimum necessary reporting standards for engagement in DHMI research.

Only 64% (14/22) of studies included in this review specified rate of uptake, defined as the number of participants randomized to the intervention condition who used the app at least once. Past research suggests that rate of uptake cannot be assumed, especially in the context of fully remote, self-guided digital interventions. Those studies that did report this metric showed varied levels of uptake. For example, Arean at al [21] found that over one-half of participants did not download their assigned app, whereas Roepke et al [38] and Watts et al [13] found that closer to one-quarter of participants did not download their assigned app. The studies reviewed here varied in the type of app and design so different rates of uptake may be expected, but the extent of inconsistent reporting was surprising.

Level of use metrics, defined as both the number of app launches and the amount of time the intervention was used, was only reported in 59% (13/22) of the studies reviewed. These metrics—specifically, average number of uses and average time spent in the app—should be feasible to calculate when researchers have access to activity log data of the tested app, which was the case in most of the studies included. There can be some complications reporting these metrics. For example, it can be difficult to accurately report time spent in the app when participants leave an app open on their device for longer than they are actively using it. Similarly, apps can be launched only to be closed in a matter of seconds. However, in cases where these metrics are not appropriate for the intervention being evaluated, we would have expected to see alternative metrics such as number of clicks reported, but this was only the case in 1 of the reviewed studies [36].

Approximately one-quarter of studies (5/22, 23%) reported on participant duration of use, defined as reporting both weekly use patterns and the number of participants who used the app in the final week of the intervention period. It is well documented that, in general, mobile apps tend to be used heavily when first downloaded and that use decreases over time [41]. Similarly, concerns related to sustained engagement with web-based psychiatric interventions have been reported in routine-care implementation studies [4-6,17]. Inconsistent use of psychiatric intervention apps over time is an issue that needs to be addressed if our field is to mature; however, addressing this issue will be all the more difficult if such variations in use are not adequately reported in our published literature. Data from Dahne et al [25] provide an excellent example of how this metric is useful to report alongside level of use. They reported that 81.8% of participants in the intervention condition used the app at least eight times (an average of at least once per week), but only 51% of participants used the app during the last week. Much like patterns of use with other popular apps, these data suggest high initial use that declines over time.

In the context of intervention research, it is important to include some clear metric of intervention adherence or completion. Yet only 50% (11/22) of studies in this review clearly reported the number of participants considered to have completed the app-based components of the intervention as intended or other metrics related to completion such as percentage of patients who met a specified adherence threshold; percentage of patients completing clinician-based components of the intervention; and percentage of participants who downloaded all the intervention content. Just like psychotherapy or medication use, mobile app–based interventions incorporate some expected efficacious dose into the instructions for use. The fact that use can be accurately and objectively tracked from backend metrics is highly encouraging, and distinguishes our field from other treatment research (such as medication trials) where adherence has historically been extremely difficult to reliably measure. Further, completion need not be full use exactly as intended. For example, Arean et al [21] specified that 50% compliance with intervention instructions was considered completion. Simply not discussing who uses mobile app depression interventions as intended, however, will limit the potential for insight into and utility of these interventions.

Finally, one of our objectives in this review was to quantify standard level of engagement in RCTs of mobile app–based depression interventions. Our data extraction led us to conclude that with the current state of reporting, this is nearly impossible to do. What we did conclude is that engagement at all points—uptake, level, duration, and completion—is widely varied. Moreover, it was not uncommon to see completion rates at or below 50% of those participants randomized to a treatment condition (n=6) or to simply see engagement rates not reported at all (n=5).

### Limitations

This scoping review has several limitations. First, this review illustrates an important dilemma in the field of DMHI research, but findings are limited to a subset of DMHI literature, specifically only that involving depression interventions in psychiatric samples with mobile app–based interventions. While we expect our proposed reporting guidelines to be useful across DMHIs, the extent to which the findings of this review carry through to mobile app interventions in other areas of mental health remains unclear. Second, our original goal in approaching this scoping review was to quantify typical engagement with DMHIs in RCTs; however, as we began the literature review, we ascertained that this goal would be difficult given the variability (and often absence) of metrics reported. This study, therefore, represents a shift in objectives. Third, we only reviewed papers from academic sources, which limits the kinds of mental health apps we took into account. The quality and objectivity of the data contained within independently published reports from private industries on their own mental health apps have yet to be reviewed. Finally, this review only evaluated literature though May 2020. While there is no reason to expect that reporting on engagement has improved, this work should be conceptualized as only a starting point for a discussion of appropriate reporting guidelines and future reviews or meta-analyses on this topic are warranted.

### Conclusions

The emerging field of DMHIs has reached a critical juncture: intervention engagement has been widely recognized as the key factor limiting DMHI clinical utility. This review illustrates that engagement is variable and frequently underreported. Adopting a set of reporting guidelines that specify the minimum necessary information when publishing RCTs of DMHIs will provide new insights into how to improve engagement in mental health apps; allow for clear comparisons between DMHIs and other treatment options; and offer benchmarks upon which further research must improve. Such reporting standards will complement the expanding literature on user-centered evaluations of engaging with digital health tools and interventions [42-44].

To this end, we suggest the 5-element framework applied in this study be used to guide minimum necessary DMHI engagement reporting standards. This framework includes the following: (1) intervention instructions or adherence criteria, defined as an explicit statement of what it means for participants to have used an intervention as intended or met some minimum intervention threshold; (2) rate of uptake, defined as the number of participants randomized to the intervention who downloaded the associated app(s) and used them at least once; (3) level of use metrics, defined as *both* the number of app launches and the amount of time the intervention was used (with alternative metrics such as number of clicks appropriate if more suitable for the intervention and justified); (4) duration of use, defined as participants’ weekly use patterns; and (5) number of completers, defined as the number of participants who completed the intervention as intended per intervention instructions or per specified adherence criteria. We believe this framework could be a useful starting point to promote standards of reporting within the field, with room for future iterations.

Certainly complexities exist when identifying and reporting engagement with DMHIs given that these interventions vary widely in content and format. The reporting guidelines that we have suggested in response to our findings are intended both to be broadly applicable across DMHIs and to challenge the field to move past complexities and move toward greater transparency and rigor. We hope this begins an important discussion on reporting standards that will improve our understanding of how to evaluate and optimize DMHIs.

## Appendix

Multimedia Appendix 1 Preferred Reporting Items for Systematic Reviews and Meta-Analyses Extension for Scoping Reviews (PRISMA-ScR) Checklist.

Multimedia Appendix 2 Other DMHI use data reported. DMHI: digital mental health intervention.

## Abbreviations

AMED: Allied and Complementary Medicine
DMHI: digital mental health intervention
HMIC: Health Management Information Consortium
HTA: Health Technology Assessment
PRISMA: Preferred Reporting Items for Systematic Reviews and Meta-Analyses
RCT: randomized controlled trial
