# Supplementary material for: Digital Mental Health Interventions for Depression: Scoping Review of User Engagement
Source: J Med Internet Res. 2022 Oct 14;24(10):e39204. doi: 10.2196/39204 (PMC9617183; doi:10.2196/39204)
Supplement: Multimedia Appendix 2 [file jmir_v24i10e39204_app2.docx]

Appendix 2. Other DMHI Use Data Reported

| **Study** | **Intervention Name** | **Other App Use Engagement Metrics** |
| --- | --- | --- |
| Arean et al. 2016^21^ | Project: EVO app | None |
|  | iPST | None |
|  | Health Tips app | None |
| Bakker et al. 2018^22^ | MoodKit | None |
|  | MoodPrism | None |
|  | MoodMission | None |
| Birney et al. 2016^23^ | MoodHacker | None |
| Borjalilu et al. 2019^24^ | Aramgar app | None |
|  | Aramgar app with face-to-face therapy | None |
| Dahne et al. 2019^25^ | ¡Apívate! | - Mean seconds per session was 87.73 (SD = 63.08) - 81.8% of participants used the app at least 8 times (i.e., at least once per week on average), 45.5% of participants used the app at least 28 times (i.e., every other day on average), and 36.4% of participants used the app 56 or more times (i.e., at least once per day on average) - Participants created on average 4.68 (SD = 3.54) unique values within the app, 11.77 (SD = 17.02) activities across values, and completed 21.73 (SD = 45.60) activities |
|  | iCouch CBT | - Percentage of participants utilizing the app at least once a week was lower than !Apitivate!, with significant differences observed at week 2 (p<.01) and week 3 (p<.05) |
| Dahne et al. 2019^26^ | Moodivate | - 71.4% of participants used the app at least 28 times, and 42.9% of participants used the app more than 56 times (i.e., at least once per day on average) - Spent an average of 3.50 (SD = 2.76) minutes using the app per session - Participants created on average 6.10 (SD = 3.22) unique values within the app, 14.71 (SD = 10.22) activities across values, and completed 52.24 (SD = 89.31) activities |
|  | MoodKit | None |
| Faurholt-Jepsen et al. ^15^ | MONARCA | - On average 85 text messages were sent and 11 phone calls were made to each patient, and all patients had contact with the study nurse during the trial period (appears to refer to both groups) |
|  | Placebo MONARCA |  |
| Fitzpatrick et al. 2017^27^ | Woebot | None |
| Guo et al. 2020^28^ | Run4Love | - Participants completed on average 55% of the cognitive behavioral stress management coursework at 3 months |
| Ludtke et al. 2018^29^ | Be Good to Yourself | None |
| Ly et al. 2014^30^ | Behavioral Activation smartphone app | - Across groups, therapists reported 2-18 minutes per week of time commitment per participant |
|  | Mindfulness smartphone app |  |
| Ly et al. 2015^31^ | Blended Treatment | - Average time in session was 240 minutes (compared to 600 minutes in the full behavioral activation group) - Average time therapist spent in the back-end system was 80.7 minutes (SD = 41.6) |
| Mantani et al. 2017^32^ | CPT-Kokoro-App | - Average of 10.8 (SD = 4.2) days to complete one session - Mean 11.2 (SD = 11.4) “mind maps” for self-monitoring completed - Mean 14.4 (SD = 17.1) behavioral activation tasks completed - Mean 6.1 (SD = 6.0) alternative thoughts for cognitive restructuring completed - N = 16 participants discontinued protocol antidepressant treatment during the study (compared to N=14 in the antidepressant switch treatment group) |
| Moberg et al. 2019^33^ | Pacifica | None |
| Mohr et al. 2019^34^ | IntelliCare - Coached | - Participants who did not receive coaching downloaded a median of 7 apps (IQR 4-10) and those who received coaching downloaded a median of 11 aps (IQR 10-12). - Median time to last use of any app was 56 days (IQR 54-56) and there was no significant difference across groups in time to last engagement - There was a significant effect of receiving recommendations on number of app sessions - After the trial, 253 (84%) of participants continued using the IntelliCare apps. Among those who continued to use the apps, the median time from end of treatment to last use was 92 days (IQR 14-178) with a median of 83 (IQR 11-286) sessions. |
|  | IntelliCare - Self-guided |  |
|  | IntelliCare -Recommendations |  |
|  | IntelliCare - No Recommendations |  |
| Motter et al. 2019^35^ | Executive Function / Professing Speed Focused CCT | None |
|  | Verbal Ability Focused CCT | None |
| O'Toole et al. 2019^36^ | LifeApp’tite | - Mean number of total clicks (for 50 active participants) was 297.9 (SD = 180.3) - 23 (38.3%) used the methods library and the mean number of methods used among those using the library was 4.0 (SD = 2.6) - Average number of sessions with a study clinician was 9.4 (SD = 4.1) compared to 10.4 (SD = 5.2) in the TAU condition |
| Place et al. 2020^37^ | Cogito | None |
| Proudfoot et al. 2013^40^ | MyCompass | - The mean number of modules completed was 1.6 (SD = 1.7; range 0 to 9). - Participants self-monitored an average of 49 times (SD = 54.1; range 0 to 262) |
| Roepke et al. 2015^38^ | CBT-PPT SuperBetter | - 31 (33.3%) downloaded all content - 68 participants (73.1%) downloaded some content - 37 participants (39.8%) downloaded PPT content only |
|  | General SuperBetter | - 64 (66.0%) downloaded all content |
| Stiles-Shields et al. 2018^39^ | Boost Me | - Number of events logged in Boost Me was 14.7 ± 10.1 - Mean clinician call duration was 6.20 ± 3.30 minutes |
|  | Thought Challenger | - Number of events logged in Thought Challenger was 8.5 ± 11.6 - Mean clinician call duration was 4.27 ± 0.83 minutes |
| Watts et al. 2013^13^ | Get Happy Program Mobile App | - Aggregated mean score for homework effort was 11.25 (range 6-17) - Aggregated mean score for homework completion was 11 (range 8-16) - Across groups mean clinician time spent per participant was 4.1 minutes (SD = 4.63) |

*Note*. Table includes all treatment conditions that involved a mobile app component. SD = standard deviation; IQR = interquartile range; TAU = treatment as usual.
